# Targeted lymphodepletion with a CD45-directed antibody radioconjugate as a novel conditioning regimen prior to adoptive cell therapy

**DOI:** 10.18632/oncotarget.27731

**Published:** 2020-09-29

**Authors:** Wojciech Dawicki, Kevin J.H. Allen, Ravendra Garg, Eileen M. Geoghegan, Mark S. Berger, Dale L. Ludwig, Ekaterina Dadachova

**Affiliations:** ^1^College of Pharmacy and Nutrition, University of Saskatchewan, Saskatoon, SK, Canada; ^2^Actinium Pharmaceuticals, New York, NY, USA

**Keywords:** anti-CD45 targeted lymphodepletion, radioimmunotherapy, adoptive cell therapy, 131Iodine, 177Lutetium

## Abstract

Chimeric antigen receptor (CAR) T cell therapies, and adoptive cell therapy (ACT) in general, represent one of the most promising anti-cancer strategies. Conditioning has been shown to improve the immune homeostatic environment to enable successful ACT or CAR-T engraftment and expansion *in vivo* following infusion, and represents potential point of intervention to decrease serious toxicities following CAR-T treatment. In contrast to relatively non-specific chemotherapy-derived lymphodepletion, targeted lymphodepletion with radioimmunotherapy (RIT) directed to CD45 may be a safer and more effective alternative to target and deplete immune cells. Here we describe the results of preclinical studies with an anti-mouse CD45 antibody 30F11, labeled with two different beta-emitters 131Iodine (^131^I) and 177Lutetium (^177^Lu), to investigate the effect of anti-CD45 RIT lymphodepletion on immune cell types and on tumor control in a model of adoptive cell therapy. Treatment of mice with 3.7 MBq ^131^I-30F11 or 1.48 MBq ^177^Lu-30F11 safely depleted immune cells such as spleen CD4+ and CD8+ T Cells, B and NK cells as well as Tregs in OT I tumor model while sparing RBC and platelets and enabled E. G7 tumor control. Our results support the application of CD45-targeted RIT lymphodepletion with a non-myeloablative dose of ^131^I-30F11 or ^177^Lu-30F11 antibody prior to adoptive cell therapy.

## INTRODUCTION

Chimeric antigen receptor (CAR) T cell therapies, and adoptive cell therapy (ACT) in general, represent one of the most promising anti-cancer strategies [1]. To date, two autologous CAR-T cell therapies directed against CD19 have been approved for use in the treatment of B-cell lymphomas including relapsed or refractory acute lymphoblastic lymphoma (ALL) and diffuse large cell B-cell lymphoma (DLBCL) [2]. Initial response rates in these heavily pretreated patients have been extraordinary, in the range of 80%, although durable responses have been considerably lower at 40–50% [2]. There are now nearly 1000 clinical trials involving CAR-T cells in the US (https://clinicaltrials.gov/), including expanding to new targets such as BCMA and CD123 in heme malignancies and various targets in solid tumors. Further, recombinant T cell receptor (TCR) engineered T cells, TCR-T that are directed against MHC-complexed peptides, are also being evaluated primarily in solid tumors [3].

It is unclear why some patients respond to treatment with adoptive cell therapies such as CAR-T, and others do not, though the tumor immune microenvironment is a likely contributor to variable effect of cell therapy in both hematologic and solid cancers. To this end, preclinical and clinical studies have shown that regulatory T cells (Tregs) can have an impact on the effect of ACT in mice and in patients with melanoma [4, 5]. In these studies, depletion of Tregs, whether by specific depletion or via conditioning with external beam radiation, had a favorable impact on the anti-tumor response to ACT. Interestingly, these and other studies suggest that Treg depletion is more sustained following treatment with radiation as opposed to chemotherapy-induced conditioning [6], where a rapid rebound of Tregs was seen with chemotherapy conditioning resulting in poorer outcomes. Other cell types that contribute to an immunosuppressive tumor microenvironment that may negatively impact CAR-T efficacy include myeloid derived suppressor cells (MDSCs) and tumor-associated macrophages (TAMs) [7].

Another important area for improvement in CAR-T therapies involves the serious adverse events that have been reported, particularly with the drugs directed against CD19 such as tisagenlecleucel and axicabtagene ciloleucel. Cytokine release syndrome (CRS) and immune effector cell-associated neurotoxicity syndrome (ICANS) occur at very high rates following CD19 CAR-T administration, with CRS occurring in greater than 50% of patients and at least 10–30% patients experiencing high grade ICANS [2]. Recent preclinical studies have shown that cytokine release leading to CRS or neurotoxicity is due to activated macrophages following recruitment to the site of CAR-T and tumor cells. Mouse study results [8, 9] documented that macrophages secrete IL-1 or IL-6 following recruitment and activation by CAR-T cells at the tumor site.

Conditioning has been shown to improve the immune homeostatic environment to enable successful ACT or CAR-T engraftment and expansion *in vivo* following infusion, and represents a potential point of intervention to decrease serious toxicities following CAR-T treatment. Most CAR-T programs exploit the use of the combination of fludarabine and cyclophosphamide (flu/cy) as a lymphodepletive conditioning regimen prior to CAR-T. These drugs are often administered 2–7 days (2–5 day course of therapy) prior to ACT infusion. However, the commonly used flu/cy regimen is a non-specific and cytotoxic treatment that some patients may not be able to tolerate and may not offer tumor control. Additionally, flu/cy has been correlated with toxicities such as prolonged cytopenias and cytokine release syndrome (CRS) following CAR-T administration [10].

In contrast to relatively non-specific chemotherapy-derived lymphodepletion, targeted lymphodepletion with radioimmunotherapy (RIT) directed to CD45 may be a safer and more effective alternative to target and deplete immune cells. The CD45 antigen is found on all nucleated immune cells, with increased expression on mature lymphoid and myeloid lineages, leading to preferential depletion of mature immune cells compared to progenitor hematopoietic cells [11]. Importantly, immunomodulatory cells such as Tregs and MDSC express CD45 and are targets of lymphodepletion with a CD45-targeting antibody-radionuclide conjugate (ARC), potentially resulting in better engraftment, activation and persistence of the exogenously added CAR-T cells in patients. In addition, macrophages, implicated in CRS, are also sensitive to targeting with a CD45 ARC, and their transient reduction may result in mitigation of CRS risk. In addition, most hematologic malignancies such as leukemia and lymphoma abundantly overexpress CD45, at levels of 200 to 400,000 antigens per cell. Targeted lymphodepletion with a CD45 ARC is anticipated to result in a reduction in tumor burden, which may result in an improvement in overall response to the CAR-T therapy.

Anti-CD45 RIT with 131Iodine (^131^I)-apamistamab (Iomab-B), is in a Phase III clinical trial as a myeloablative targeted conditioning regimen prior to allogeneic stem cell transplant in patients with active relapsed/refractory acute myeloid leukemia (AML). Results from patients following dosimetry testing have shown that low non-myeloablative doses of ^131^I-apamistamab were able to safely induce transient lymphodepletion [12]. This data allowed us to hypothesize that low dose anti-CD45 RIT could be used as a targeted modality to effectively lymphodeplete prior to ACT. Here we describe the results of preclinical studies with an anti-mouse CD45 antibody 30F11, labeled with two different beta-emitters - ^131^I and 177Lutetium (^177^Lu), to investigate the effect of anti-CD45 RIT lymphodepletion on immune cell types and on tumor control in a model of adoptive cell therapy. Our results support CD45 targeted RIT lymphodepletion prior to adoptive cell therapy using a non-myeloablative dose of ^131^I-30F11 or ^177^Lu-30F11 antibody.

## RESULTS

### ^131^I-30F11 treatment transiently depleted CD45-expressing immune cell subsets in healthy mice

**Figure 1:**
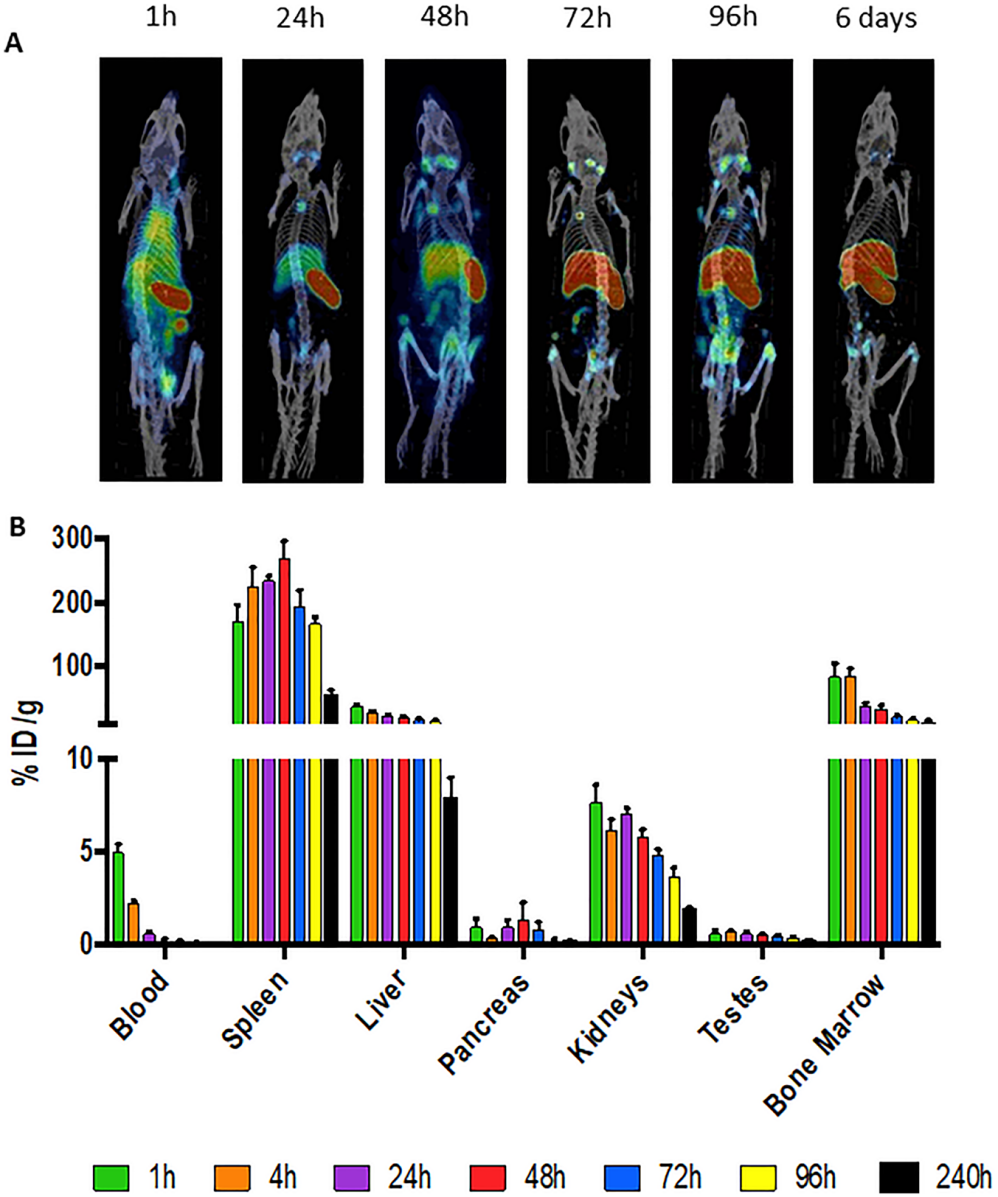
^111^In-30F11 anti-CD45 antibody homes to immune organs. (**A**) C57Bl/6 mice were injected intraperitoneally (i.p.) with 60 μg ^111^In-30F11 antibody with a specific activity of 0.185 MBq/μg and antibody distribution was monitored by microSPECT/CT at 1, 24, 48, 72, 96 hours timepoints and then again at 6 days after antibody administration. 30F11 antibody homed to immune organs: lymph nodes, spleen, and bone marrow, it also homed to the liver. (**B**) pharmacokinetics of ^111^In-labeled 30F11 in male C57Bl/6 mice.

**Figure 2:**
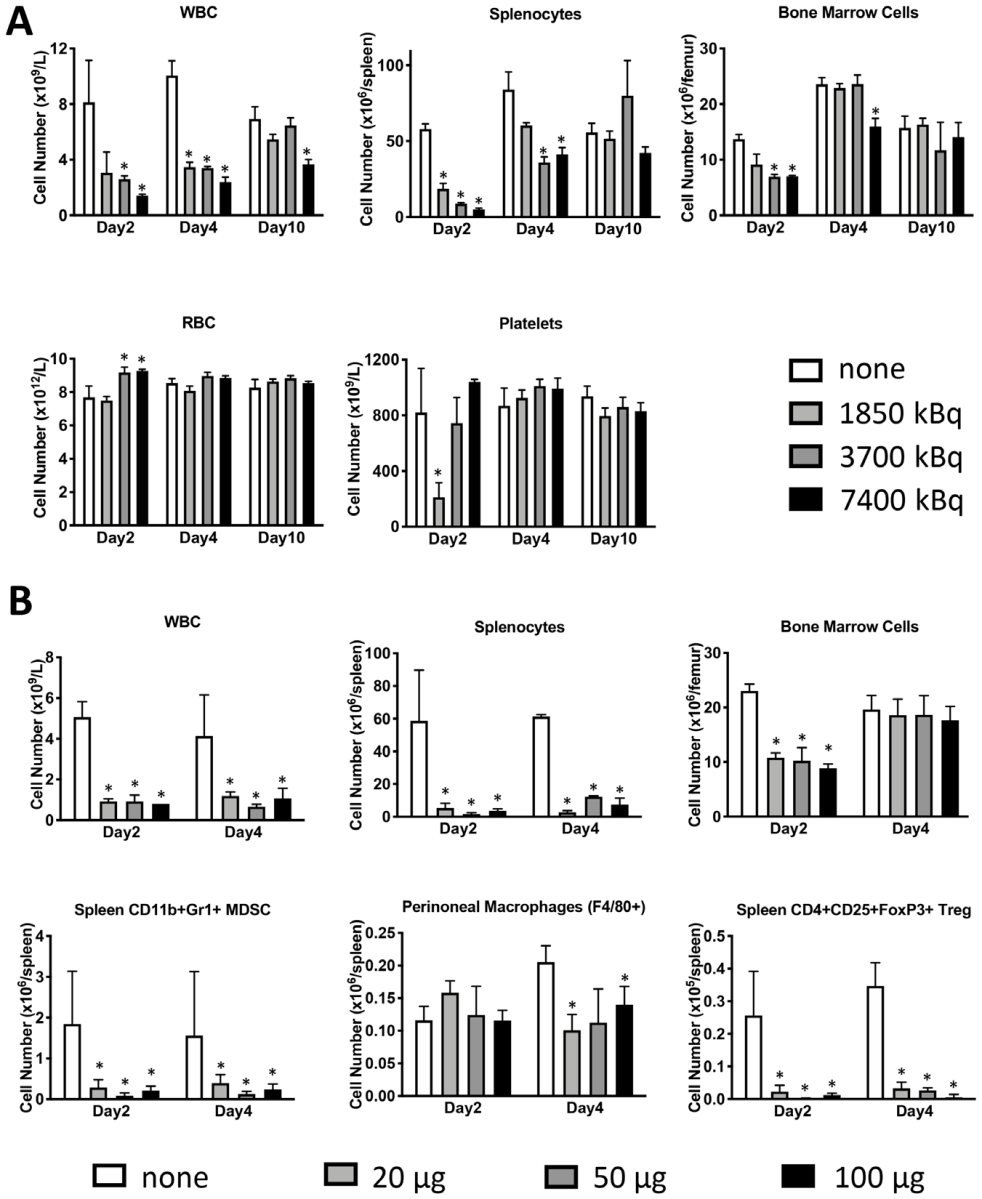
Lymphodepletion with ^131^I-labeled anti-CD45 30F11 antibody preserves bone marrow cells, platelets, and red blood cells but depletes splenocytes, Tregs and MDSC. (**A**) Dose finding studies were performed to determine the appropriate dose of ^131^I needed to define a non-myeloablative dose to safely lymphodeplete. Female C57Bl/6 mice were injected i.p. with 20 μg 30F11 labeled with 1.85-7.4 MBq ^131^I to determine the most effective and safe radioactivity dose required for transient lymphodepletion. Mice were euthanized at varying time points (2–21 days) and various immune cell subsets were quantified using flow cytometry and (**B**) To determine the appropriate amount of antibody for labeling - varying amounts of 30F11 antibody were administered to a mouse in 20–100 μg range, but with a constant activity of 3.7 MBq. Asterisks signify *P* values < 0.05.

**Figure 3:**
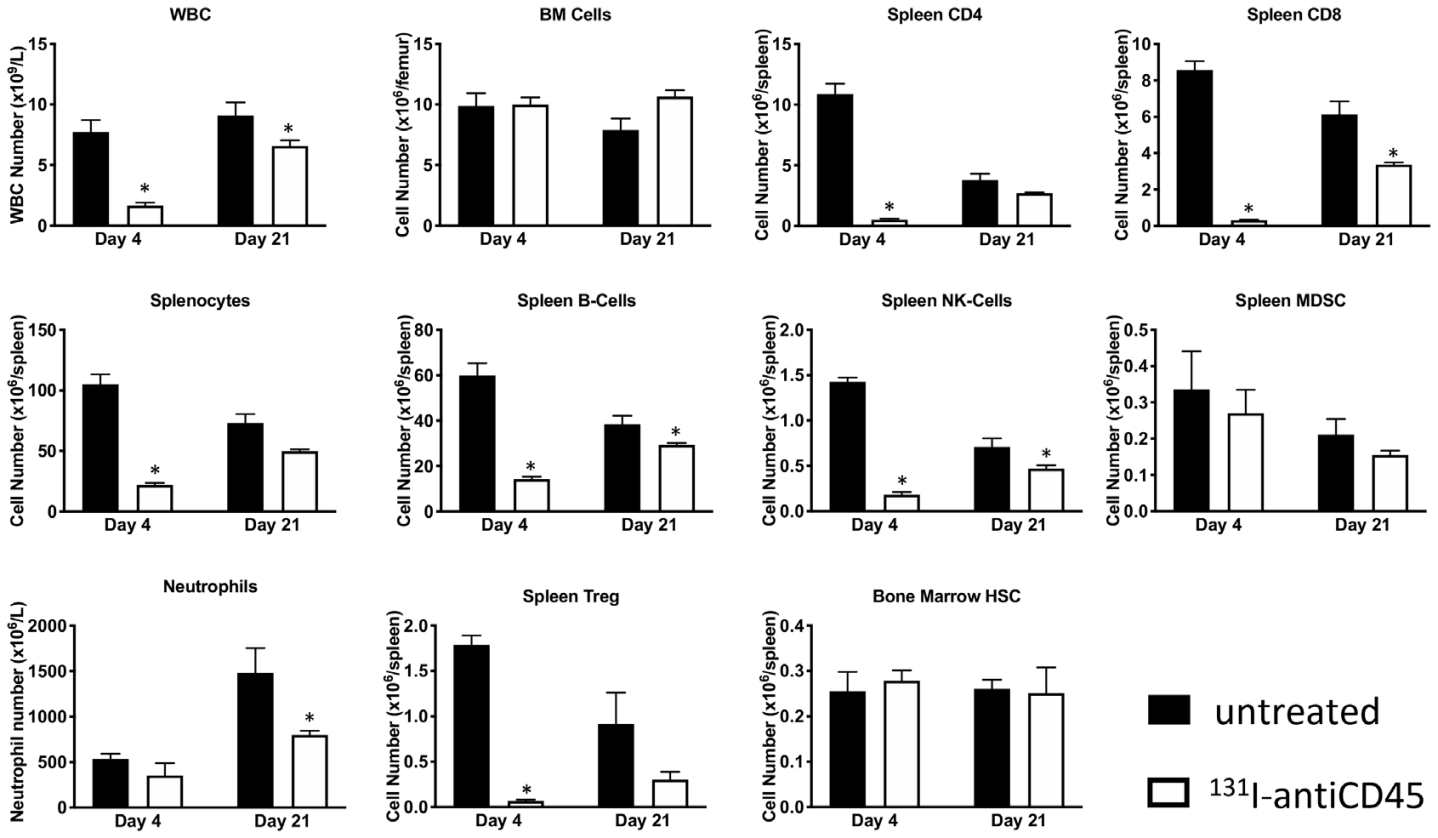
Treatment with ^131^I-labeled anti-CD45 30F11 antibody transiently depletes CD45-expressing immune cell subsets. Treatment of non-tumor bearing C57B/6 mice with 3.7 MBq ^131^I-30F11 antibody was effective in transiently lymphodepleting various lymphocyte populations such as WBC, CD4+ cells, CD8+ cells, splenocytes, B cells, MDSC, NK cells, neutrophils and Tregs at a dose that does not impact bone marrow HSCs. Asterisks signify *P* values < 0.05.

microSPECT/CT imaging of mice administered CD45-targeting antibody 30F11 radiolabeled with ^111^In (^111^In radiolabel was used in these experiments as imaging surrogate for therapeutic radionuclides ^131^I and ^177^Lu) showed that the antibody homed to immune system organs such as lymph nodes, spleen, and bone marrow as well as liver (Figure 1A). The imaging data was confirmed by the pharmacokinetics data which also demonstrated fast clearance of the antibody from the blood and kidneys, and low uptake in the pancreas and gonads (Figure 1B). Dose finding studies using 1.85–7.4 MBq ^131^I-30F11 antibody were performed next to determine the appropriate dose of ^131^I needed to define a non-myeloablative dose to safely lymphodeplete. Figure 2A shows that 3.7 MBq dose of ^131^I-30F11 transiently depleted lymphocytes, splenocytes, and myeloid derived cells (MDSC) but preserved bone marrow cells, platelets, and red blood cells. Experiments in which variable amounts of 30F11 were radiolabeled with 3.7 MBq ^131^I revealed no effect of the antibody amount on the efficacy of lymphodepletion (Figure 2B). Based on these results, 20 μg of antibody was labeled with 3.7 MBq ^131^I for targeted lymphodepletion in the follow-up experiments. Importantly, the detailed analyses of the depleted cells subpopulations showed that ^131^I-30F11 was able to deplete subsets such as spleen NK and B cells, neutrophils and spleen Tregs at a dose that did not impact bone marrow hematopoietic stem cells (HSCs) (Figure 3).

### ^131^I-30F11 treatment safely depletes immune cells in OT I tumor model and enables tumor control

**Figure 4:**
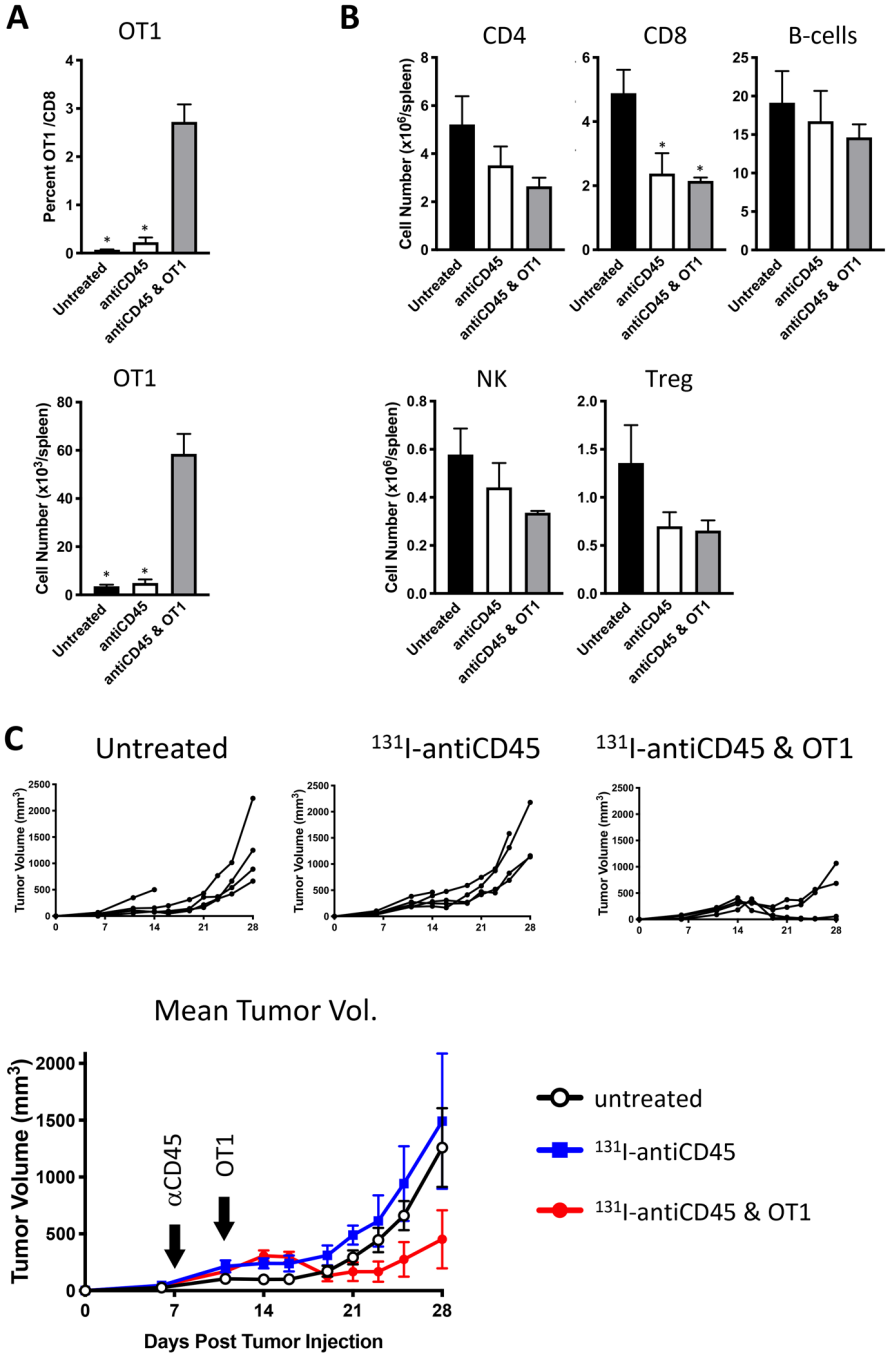
Lymphodepletion with ^131^I-labeled anti-CD45 30F11 antibody enables tumor control in OT I adoptive cell therapy model. Following E.G7 tumor engraftment, mice were conditioned with 3.7 MBq ^131^I-30F11; or received 3.7 MBq ^131^I-30F11 followed by 10^6^ OT I CD8 CD45.2 OVA reactive T cells on day 4. Control mice received no conditioning or T cells. (**A**) Targeted lymphodepletion with ^131^I-30F11 antibody enables engraftment and persistence of adoptively transferred CD45.2 OT I CD8+ T cells in the spleen 17 days post injection. (**B**) Similar to conditioning in non-tumor bearing mice, lymphodepletion with ^131^I-30F11 mediated decreases in multiple lymphoid cell subsets. (**C**) ^131^I-30F11 mediated targeted conditioning and adoptively transferred OT I T cells enabled control of E.G7 tumor growth. In 3.7 MBq ^131^I-30F11 plus OT I T cells group 2/4 mice achieved CR; in 3.7 MBq ^131^I-30F11 alone group 0/5 mice achieved CR; in untreated control group, 0/5 mice achieved CR. *N* = 4–5 per group. Asterisks signify *P* values < 0.05.

Following E.G7 tumor engraftment, mice were conditioned with 3.7 MBq ^131^I-30F11 and received 10^6^ OT I CD8 CD45.2 OVA reactive T cells on day 4. Figure 4A shows that targeted lymphodepletion with ^131^I-30F11 antibody enabled engraftment and persistence of adoptively transferred CD45.2 OT I CD8+ T cells in the spleen 17 days post injection. Similar to conditioning in non-tumor bearing mice, ^131^I-30F11 lymphodepletion mediated decreases in multiple lymphoid cell subsets such as spleen CD4+, CD8+, B and NK cells as well as Tregs. (Figure 4B). Importantly, while ^131^I-30F11 mediated targeted conditioning did not affect the tumor growth – the combination of ^131^I-30F11 mediated targeted conditioning and adoptively transferred OT I T cells enabled control of E.G7 tumor growth (Figure 4C). In the control group which received no conditioning or T cells and in ^131^I-30F11 alone groups, 0% of mice achieved complete response (CR), while in the treated group which received immunodepletion followed by OT I cells, 50% of mice (2/4) achieved CR (*p* = 0.02).

### ^131^I-30F11 and ^177^Lu-30F11 treatment produced comparable effects on immune cells and tumor growth

**Figure 5:**
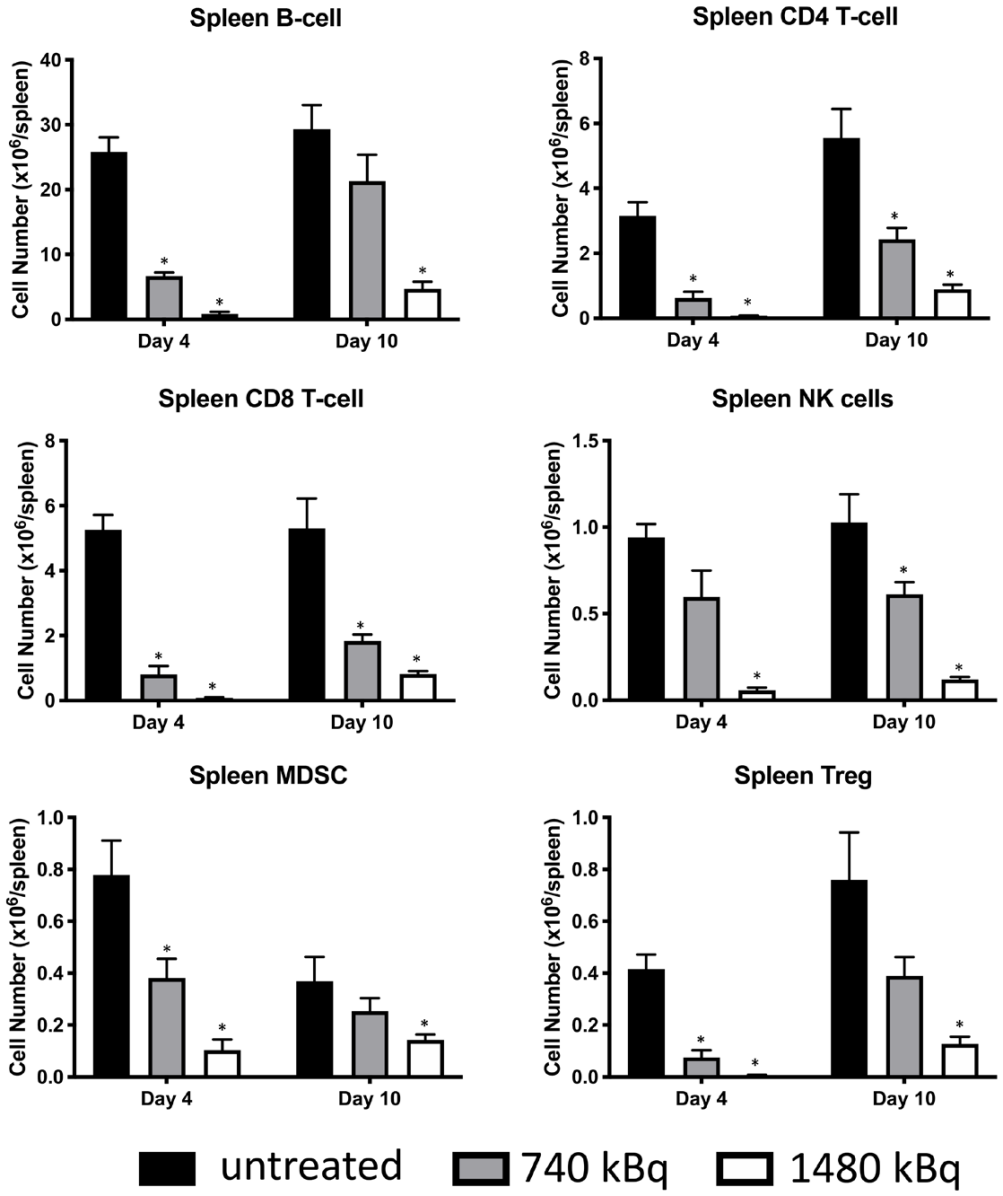
Treatment of non-tumor bearing C57B/6 mice with 1.48 MBq ^177^Lu-30F11 antibody was effective in transiently depleting various immune populations in the spleen such as CD4+ T cells, CD8+ T cells, B cells, MDSC, NK cells and Tregs. Asterisks signify *P* values < 0.05.

**Figure 6:**
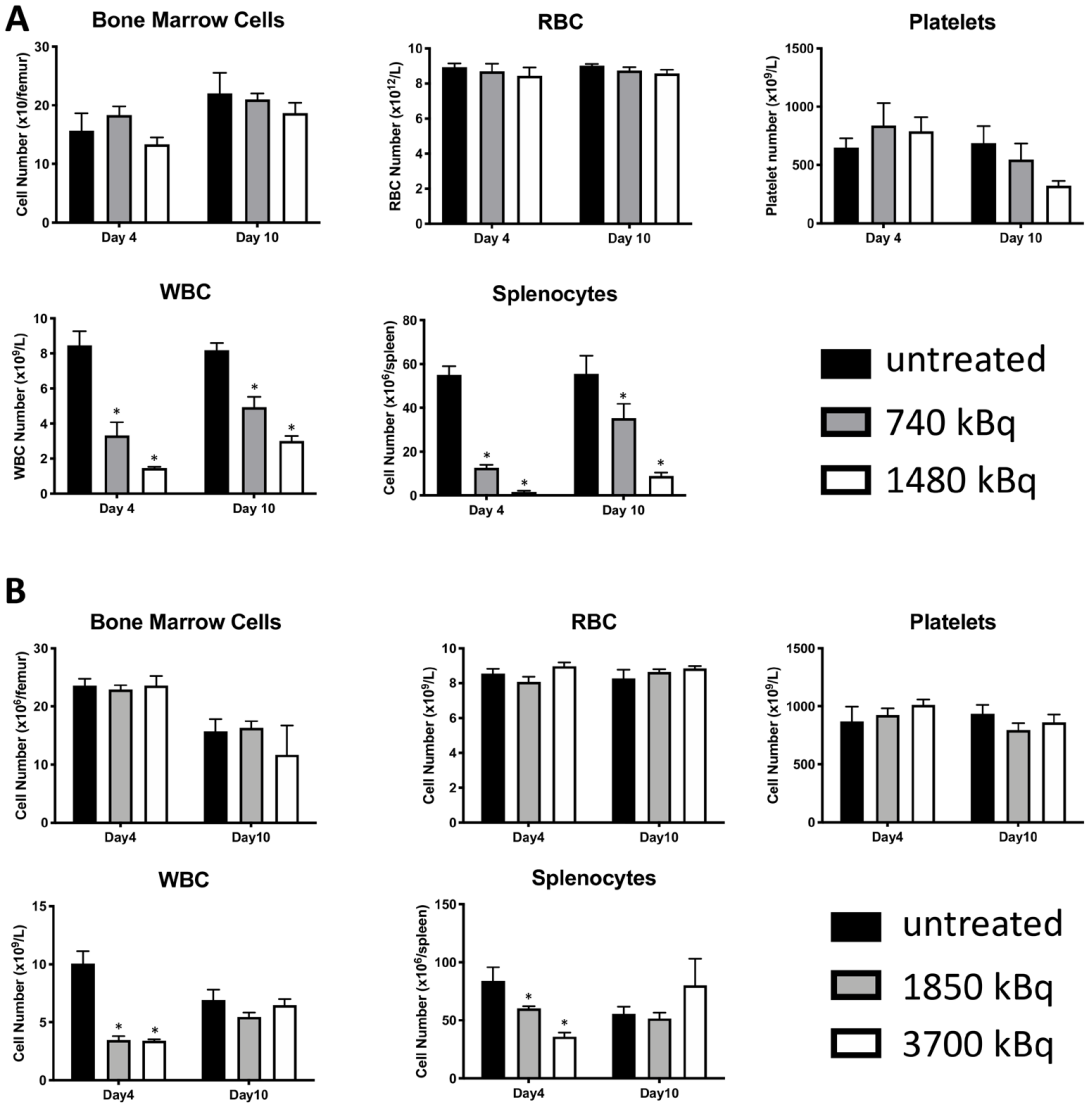
^177^Lu-30F11 or ^131^I-30F11 antibody transiently depleted CD45+ immune cell subsets without affecting platelets, red blood cells, or bone marrow cells. Treatment of non-tumor bearing C57B/6 mice with (**A**) 0.74 or 1.48 MBq ^177^Lu-30F11; (**B**) 1.85 or 3.7 MBq ^131^I-30F11 antibody was similarly effective in transiently lymphodepleting various immune cell populations without affecting bone marrow cells, red blood cells, and platelets. Asterisks signify *P* values < 0.05.

**Figure 7:**
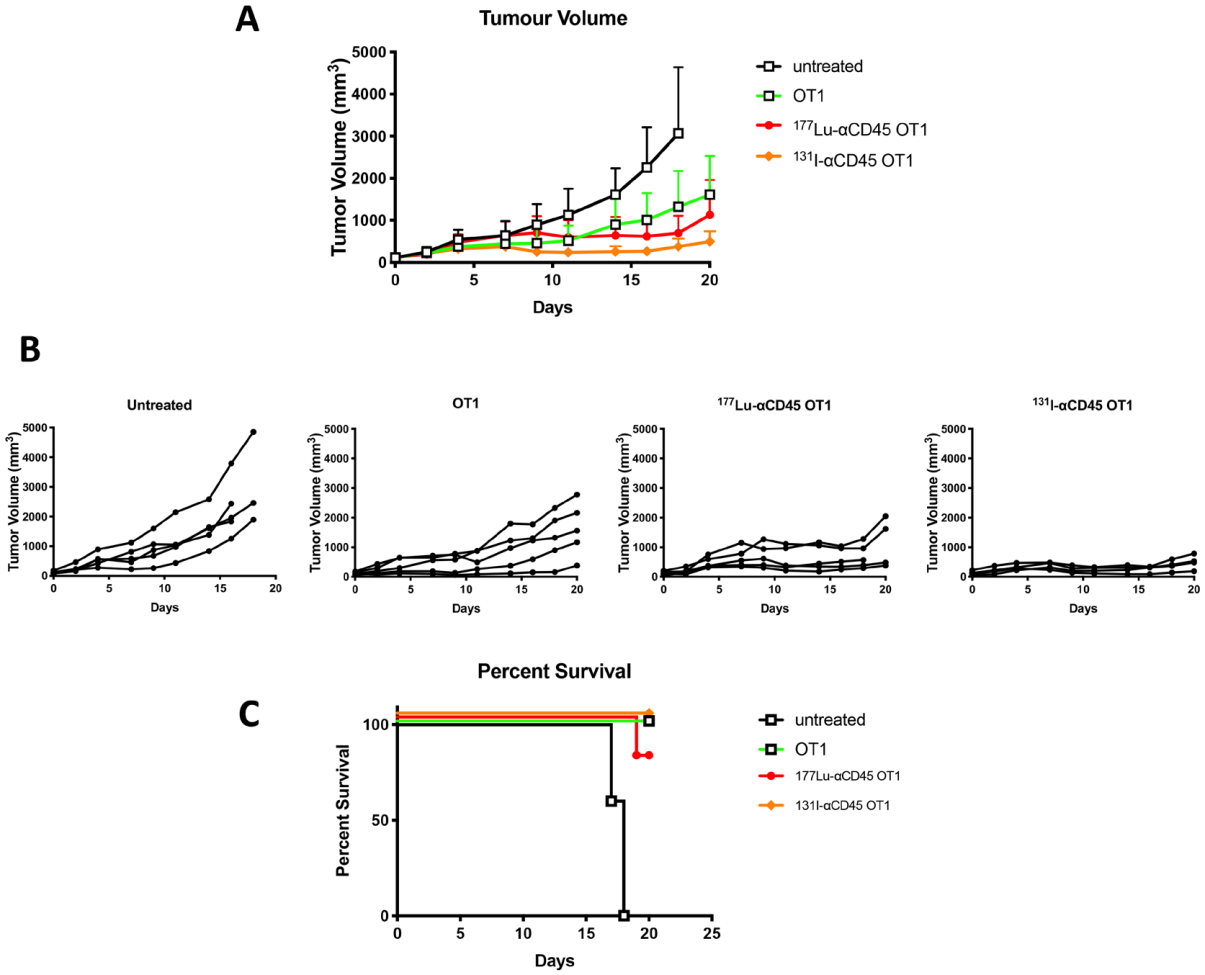
Comparative tumor control by ^177^Lu-30F11 or ^131^I-30F11 antibody. Following E. G7 tumor engraftment, mice either received no treatment or were conditioned with 1.48 MBq ^177^Lu-30F11 or 3.7 MBq ^131^I-30F11 on Day 0 and then received 10^6^ OT I CD8+ CD45.2 OVA reactive T cells on day 4. (**A**) ^177^Lu-30F11 and 131I-CD45-mediated targeted conditioning prior to adoptively transferred OT I T cells enabled control of E.G7 tumor growth. (**B**) Tumor size for individual mice in each group is displayed. OT I T cell persistence and expansion was confirmed in mice at the time of sacrifice. (**C**) Survival of mice in treated and in untreated control groups.

Pilot treatment of non-tumor bearing C57B/6 mice with 0.74 or 1.48 MBq ^177^Lu-30F11 antibody revealed that 1.48 MBq ^177^Lu-30F11 was effective in transiently depleting various immune populations in the spleen including Tregs (Figure 5). Subsequently, we performed side by side comparison of lymphodepletion in non-tumor bearing C57B/6 mice with either 0.74 or 1.48 MBq ^177^Lu-30F11 or 1.85 or 3.7 MBq ^131^I-30F11. Both radiolabeled molecules were similarly effective in transiently lymphodepleting various immune cell populations without affecting bone marrow cells, red blood cells, or platelets (Figure 6). The experiment interrogating the ability of ^177^Lu- and ^131^I-30F11 mediated targeted conditioning prior to adoptively transferred OT I T cells to control E.G7 tumor growth showed comparable effect of both agents on the tumor size and overall survival with 20% better tumor control and higher overall survival in ^131^I-30F11 treated group (*p* = 0.03) (Figure 7).

## DISCUSSION

Prior to a patient receiving a dose of an adoptive cell transfer such as engineered autologous or allogeneic CAR-T cells, it is common to perform a lymphodepletion step often using high dose chemotherapy [2, 6]. This process is considered important to create sufficient space in the immune microenvironment, e.g., bone marrow, to allow the transferred cells to engraft. Further, lymphodepletion appears to elicit a favorable cytokine profile for establishment and proliferation of the adoptively transferred lymphocytes [4]. In this study we demonstrate the feasibility and utility of radioimmunotherapy (RIT) using low non-myeloablative doses of ^131^I-radiolabeled anti-CD45 30F11 antibody to effectively lymphodeplete in a targeted manner in experimental models prior to administration of adoptive cell therapy. Significantly, targeted conditioning with pan-CD45 RIT, which selectively targets all nucleated immune cells, depletes not only lymphocytes, but also macrophages, as well as immune suppressive regulatory T cells and myeloid-derived suppressor cells in the immune microenvironment. It can potentially also exert a direct anti-tumor effect on CD45+ hematopoietic cancers.

Subsequently, we investigated use of an alternate payload selection, specifically ^177^Lu, for mediating lymphodepletion in mouse models. Both ^131^I and ^177^Lu are beta emitters with physical half lives of 8 and 6.6 days, respectively, and maximum beta energies of 0.6 and 0.5 MeV, respectively. The principle difference lies in the chemistry of these radionuclides, with ^131^I being a halogen and ^177^Lu – a trivalent radiometal. ^177^Lu-labeled somatostatin receptor targeting peptide (Lutathera) has been approved for treatment of neuroendocrine tumors [13], and a ^177^Lu-labeled small molecule binding to PSMA is currently in advanced stage clinical trials for treatment of metastatic prostate cancer [14]. We have selected ^177^Lu over another beta-emitting radiometal ^90^Y as it has been shown in long term follow up studies that patients treated with ^177^Lu-peptide therapy survive significantly longer than those treated with ^90^Y-peptide therapy due to more side effects from ^90^Y [15] which is a high energy beta emitter while ^177^Lu is a low energy beta emitter. We determined that 1.48 MBq ^177^Lu-30F11 could effectively deplete various immune cell subsets in mice but spare bone marrow cells, red blood cells, and platelets. In a model of adoptive cell therapy using CD45.1 OT1 mice bearing E.G7-OVA tumors, mice that received either ^131^I-30F11 or ^177^Lu-30F11 RIT-mediated lymphodepletion demonstrated enhanced tumor control over mice that did not receive lymphodepletion. Interestingly, lymphodepletion with ^131^I-30F11 resulted in somewhat greater tumor control than with ^177^Lu-30F11 (Figure 7) which might be explained by the residualizing nature of ^177^Lu which by persisting in the tumors due its residualization, can potentially kill some of the incoming adoptive T cells.

In conclusion, our data supports CD45 targeted RIT lymphodepletion with a non-myeloablative dose of ^131^I-30F11 or ^177^Lu-30F11 prior to adoptive cell therapy.

## MATERIALS AND METHODS

### Antibodies and radiolabeling

The anti-mouse pan-CD45 binding antibody 30F11 (ThermoFisher Scientific, Catalog # 14-0451-82) was used in all experiments as a surrogate for pan-human CD45 ^131^I-apamistamab (Iomab-ACT) to perform targeted lymphodepletion in mice. Radiolabeling of 30F11 antibody with ^131^I was carried out by adding 111 MBq Na^131^I to 15 μL PBS followed by 100 μg 30F11 antibody (1,111 MBq/mg) in a microcentrifuge tube. Chloramine-T (0.2 μL, 10 mg/mL concentration) was added and the solution was shaken at RT for 5 minutes. Sodium thiosulfate (22.8 μL, 0.3 mg/mL concentration) was added followed by 1 μL Sodium Iodide solution (10% w/v) and 7.5 μL of Ascorbic acid (500 mg/mL concentration). The antibody was then purified using spin filtration via an Amicon Ultra 0.5 mL centrifugal filter (30 K MW cut off, Fisher, Hampton, NH, USA). Radiochemical purity was then checked via instant thin layer chromatography (iTLC) and was greater than 99%. For radiolabeling with ^177^Lu and ^111^In (imaging surrogate of ^177^Lu) 30F11 antibody was conjugated to bifunctional chelating agent DOTA (Macrocyclics, USA) at a ratio 20:1 DOTA: Ab and then radiolabeled with ^177^Lu or ^111^In with the specific activity of 0.185 MBq /μg antibody as in [16, 17]. Immunoreactivity of the radiolabeled 30F11 antibody was evaluated by measuring binding to E.G7-OVA cells at concentrations ranging from 0.5 ng/mL to 10 μg/mL and detecting the bound antibody with flow cytometry and anti-rat IgG2b^PE^. The EC_50_ for naked and modified 30F11 was 394 ng/mL and 393 ng/mL, respectively (Supplementary Figure 1).

### MicroSPECT/CT imaging of C57Bl/6 mice with ^111^In-labeled 30F11 antibody

Female 5 weeks old C57Bl/6 mice were injected intraperitoneally (i.p.) with 60 μg ^111^In-30F11 antibody with a specific activity of 0.185 MBq/μg and antibody distribution was monitored by microSPECT/CT at 1, 24, 48, 72, 96 hours timepoints and then again at 6 days after antibody administration. A MILabs VECTor^4^ (Netherlands) microSPECT/CT scanner was used to collect images. PMOD (version 3.9, PMOD Technologies, Inc, Zürich, Switzerland) was used for comprehensive image analysis. An Extra Ultra High Sensitivity Mouse (XUHS-M) collimator for 20–350 keV range was used to collect SPECT data using spiral trajectories. MILABS reconstruction software was used to reconstruct SPECT images using both ^111^In gamma emissions (245 keV and 171 keV) on a 0.4 mm voxel grid. For visual representation of accumulation, MIP images were utilized.

### Biodistribution of 30F11 antibody

To determine the pharmacokinetics of 30F11 antibody in immunocompetent mice, a biodistribution was carried out with ^111^In-labeled 30F11. Immediately after labelling male C57bl/6 mice were injected i.v. with 1.11 MBq (30 μg) ^111^In-30F11. Five mice were euthanized at 1, 4, 24, 48, 72, 96 and 240 hours post injection, the organs were collected into pre-weighted tubes, and activity was assessed with a 2470 Wizard2 Gamma counter (Perkin Elmer, MA, USA). A standard that contained 10% of the injected dose was also read to perform decay correction. Percent injected dose per gram (%ID/g) was determined using the equation:

%ID/g = ((sample CPM)/(organ weight^*^(standard CPM^*^10)^*^radiolabeling yield)) × 100%, where CPM are counts per minute.

### Lymphodepletion studies with ^131^I-30F11

In the first series of experiments, female 5 weeks old C57Bl/6 mice were injected i.p. with 20 μg of ^131^I-30F11 labeled with 1.85–7.4 MBq to determine the most effective and safe radioactivity dose required for transient lymphodepletion. Mice were euthanized at varying time points (2–21 days) and various immune cell subsets were quantified using flow cytometry as described below. The goal of the second series of experiments was to determine the optimal amount of antibody to be used for lymphodepletion. This was accomplished by varying the amount of 30F11 antibody administered to a mouse in 20–100 μg range, but with a constant specific activity of 3.7 MBq.

### Flow cytometry

Single cell suspensions were made by passing spleens through a 70 μm cell strainer, washed three times with FACS buffer (0.01 M azide, 2% FBS, PBS), incubated with blocking antibody for 10 min at 4°C, then labeled with surface marker-specific fluorochrome-labeled antibodies (Supplementary Table 1) for an additional 20 min at 4°C and unbound Ab was washed away with FACS buffer. Cells that were probed for intracellular markers were permeabilized (Fix/Perm Buffer; ThermoFisher Scientific, Waltham, MA, USA) for 30 min, washed again with Perm buffer, incubated with blocking antibody in the same buffer for 10 min, and then labeled with marker-specific or isotype control fluorochrome-labeled antibodies for an additional 20 min. Stained cells were washed twice with Perm buffer and once with FACS buffer, and analyzed on a CytoFLEX flow cytometer (Beckman Coulter, Mississauga, ON). The data was processed using FlowJo software (Tree Star Inc., Ashland, OR). Supplementary Figure 2 shows gating scheme for the identifications of various immune cells.

### Lymphodepletion and tumor control studies with ^131^I-30F11 in OT I mouse model

Three groups of female 5 weeks old C57Bl/6 CD45.1 mice, five animals per group, were injected subcutaneously with OVA expressing CD45+ E. G7-OVA lymphoma tumor cells. Seven days post-tumor cell injection, when tumor volume reached ~100 mm^3^, two groups of mice were treated with 3.7 MBq ^131^I-30F11 (20 μg), while the third group was left untreated. Four days post-lymphodepletion, OVA-specific CD8+ T cells were purified using anti-CD8 magnetic beads from CD45.2 OT I mice according to the manufacturer’s protocol (Miltenyi Biotec, Auburn, CA). One group of mice treated with 3.7 MBq ^131^I-30F11 was given a single i. v. injection of 10^6^ CD45.2 OT I CD8+ cells. Tumor volume and body weight were monitored, and mice were sacrificed when tumor volume exceeded 4,000 mm^3^ or tumors became necrotic. Blood and spleen were assessed for immune cell subsets and presence of engrafted CD45.2 OT I cells by flow cytometry.

### Comparative lymphodepletion studies with ^131^I-30F11 and ^177^Lu-30F11

Female adolescent C57Bl/6 mice were injected i.p. with 20 μg 30F11 labelled with 0.74 or 1.48 MBq of ^177^Lu or with 1.85 or 3.7 MBq of ^131^I to determine the ability to selectively deplete immune cell subsets. Immune cell subset quantitation was performed by flow cytometry as described above.

### Comparative lymphodepletion and tumor control studies with ^131^I-30F11 and ^177^Lu-30F11 in OT I mouse model

Female 5 weeks old C57Bl/6 CD45.1 mice were injected subcutaneously with OVA expressing CD45+ E. G7-OVA lymphoma tumor cells until 100 mm^3^ tumor volume was reached. Seven days post-tumor cell injection, mice were treated with ^177^Lu-30F11 (1.48 MBq), ^131^I-30F11 (3.7 MBq), or received no lymphodepletion treatment. Four days post-lymphodepletion, OVA-reactive CD8+ T cells isolated from CD45.2 OT I mice were administered to tumor-bearing mice. The tumor volume, body weight, immune cell subsets and presence of engrafted cells were monitored as above.

### Statistical analysis

Power analysis for the *in vivo* studies was estimated using PASS version 11 (NCSS, Inc.) using simulations of different cells depletions/tumor volumes based on pilot or literature data and conservative assumptions regarding the groups treated with the radiolabeled antibodies. All simulations showed power of at least 83% with only five animals per group because of the large differences between treated and untreated animals. Thus, 5 mice per group were utilized in the *in vivo* studies. GraphPad Prism 7 was used to analyze all the data (GraphPad Software, Inc., La Jolla, CA, USA). Differences among the groups were assessed using Student *t*-tests and one-way ANOVAs for multiple comparisons. Differences were considered significant at *p* < 0.05.

## SUPPLEMENTARY MATERIALS



## Abbreviations

CAR: chimeric antigen receptor
ACT: adoptive cell therapy
Tregs: regulatory T cells
MDSCs: myeloid derived suppressor cells
TAMs: tumor-associated macrophages
CRS: cytokine release syndrome
ICANS: immune effector cell-associated neurotoxicity syndrome
RIT: radioimmunotherapy
Ab: antibody
i.p.: intraperitoneally
OVA: ovalbumin
CPM: counts per minute
