# Supplementary material for: Targeted lymphodepletion with a CD45-directed antibody radioconjugate as a novel conditioning regimen prior to adoptive cell therapy
Source: Oncotarget. 2020 Sep 29;11(39):3571–81. doi: 10.18632/oncotarget.27731 (PMC7533072; doi:10.18632/oncotarget.27731)
Supplement: Supplementary file 1 [file oncotarget-11-3571-s001.pdf]

Targeted lymphodepletion with a CD45-directed antibody radioconjugate as a novel conditioning regimen prior to adoptive cell therapy

SUPPLEMENTARY MATERIALS

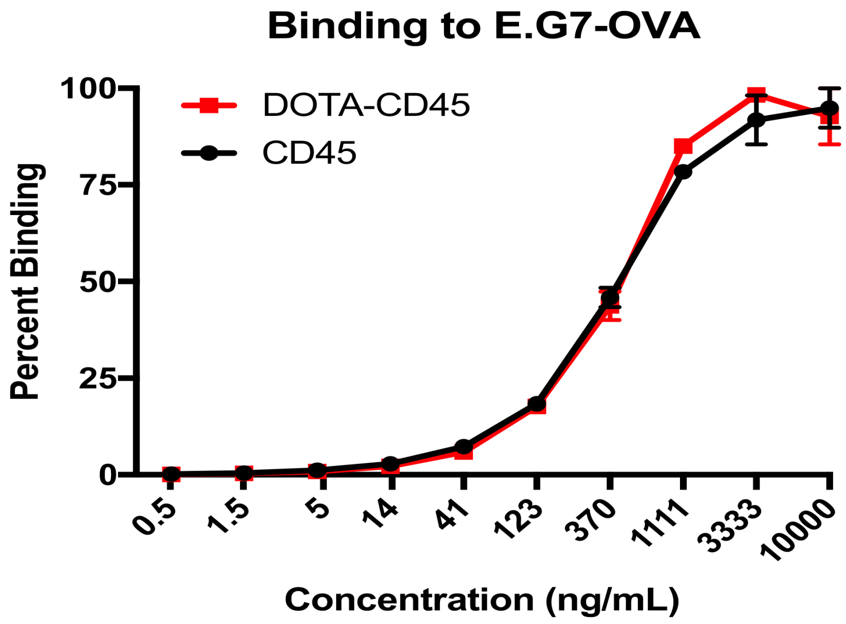

Supplementary Figure 1: Conjugation of 30F11 Ab to DOTA does not affect its immunoreactivity. Various concentrations of naked and DOTA-conjugated 30F11 Ab were incubated with E.G7-OVA cells. Bound Ab was detected with anti-rat $IgG2b^{PE}$ and analyzed by FACS. Graph represents percent of antibody binding to the cells at each concentration (experiment was performed in duplicate, mean +/– SD are shown).

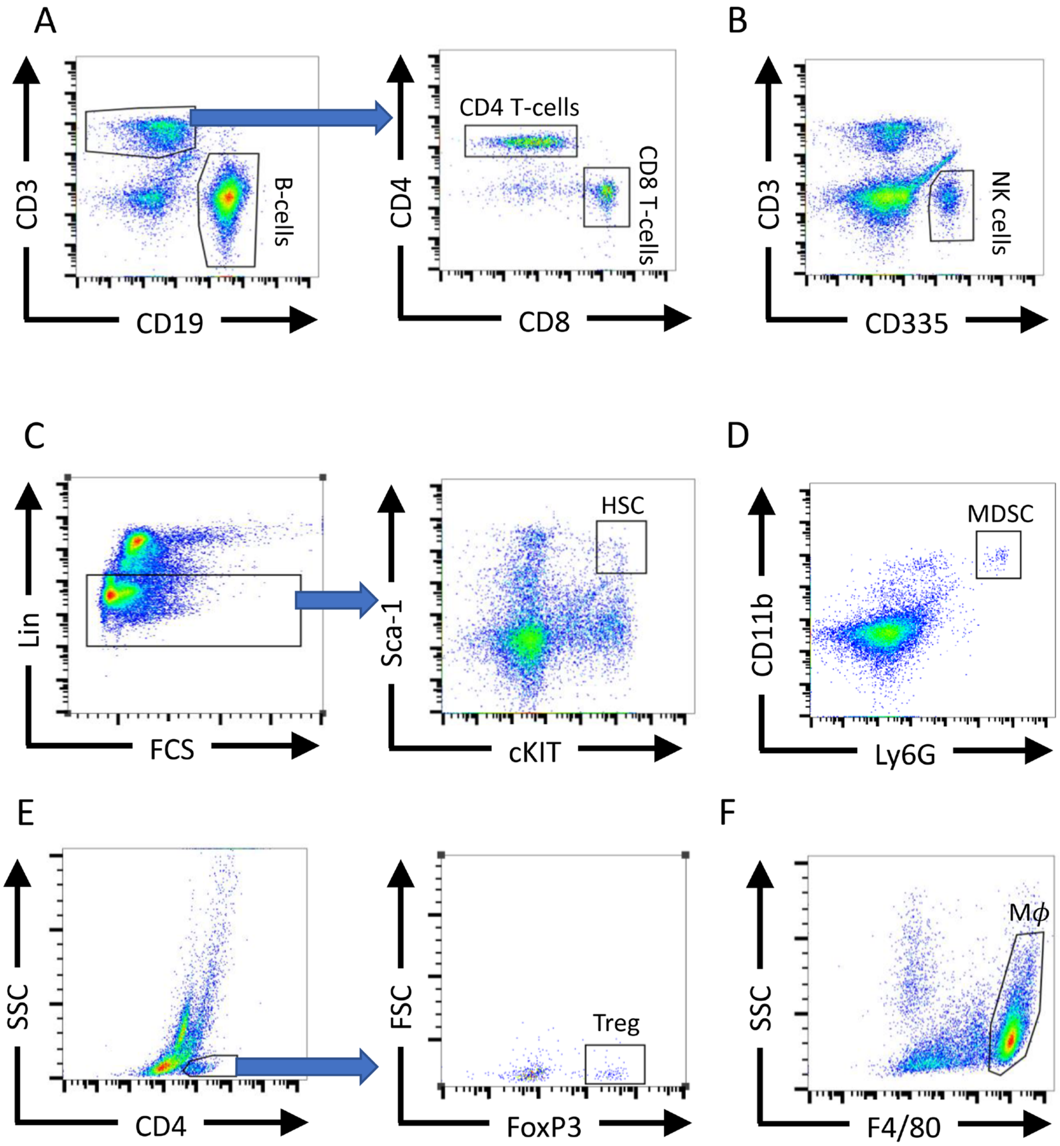

Supplementary Figure 2: Gating scheme for the identifications of various immune cells. The gating scheme for the identification of B-cells, CD4 and CD8 T-cells (**A**), NK cells (**B**), HSC (**C**), MDSC (**D**), Treg (**E**), and Macrophages (**F**). All dot plots were first gated on leucocytes using SSC and FSC and single cells using FSC-height *vs.* FSC area.

Supplementary Table 1: Antibodies used for flow cytometry

Specificity	Clone	Fluorochrome
anti-CD3 (B cells)	145-2C11	PE
anti-CD4 (T cells)	GK1.5	eFlour 450
anti-CD8 (T cells)	53-6.7	APC-eFlour780
anti-CD19 (B cells)	1D3	APC
anti-CD11b (MDSC)	M1/70	eFlour 450
anti-CD25 (Tregs)	7D4	Alexa Flour 488
anti-F4/80 (macrophages)	BM8	PE
anti-CD335 (NK cells)	NKp46	PerCP eFlour 710
anti-FoxP3 (Tregs)	FJK-16s	APC
anti-Lin	n/a	eFlour 450
anti-c-KIT (HSC)	2B8	PE
anti-SCA-1 (HSC)	D7	APC
anti-Ly6G (MDSC)	1A8-Ly6g	APC
anti-CD45.2 (splenocytes)	104	eFlour 450
anti-CD16/CD32	FRC-4G8	n/a Fc blocking
